# Odor-induced mood state modulates language comprehension by affecting processing strategies

**DOI:** 10.1038/srep36229

**Published:** 2016-10-31

**Authors:** Lin Wang, Bin Zhou, Wen Zhou, Yufang Yang

**Affiliations:** 1 CAS, Key Laboratory of Behavioral Science, Institute of Psychology, Beijing, China; 2 Jiangsu Collaborative Innovation Center for Language Ability, Jiangsu Normal University, Xuzhou, China

## Abstract

It is controversial whether mood affects cognition by triggering specific processing strategies or by limiting processing resources. The current event-related potential (ERP) study pursued this issue by examining how mood modulates the processing of task relevant/irrelevant information. In question-answer pairs, a question context marked a critical word in the answer sentence as focus (and thus relevant) or non-focus (thereby irrelevant). At the same time, participants were exposed to either a pleasant or unpleasant odor to elicit different mood states. Overall, we observed larger N400s when the critical words in the answer sentences were semantically incongruent (rather than congruent) with the question context. However, such N400 effect was only found for focused words accompanied by a pleasant odor and for both focused and non-focused words accompanied by an unpleasant odor, but not for non-focused words accompanied by a pleasant odor. These results indicate top-down attentional shift to the focused information in a positive mood state and non-selective attention allocated to the focused and non-focused information in a less positive mood state, lending support to the “processing strategy” hypothesis. By using a novel approach to induce mood states, our study provides fresh insights into the mechanisms underlying mood modulation of language comprehension.

Mood is a general and pervasive affective state^1^ that is not triggered by or directed to a specific stimulus or event. Although it is more vague and less intensive relative to emotion and usually characterized by a positive or negative valence, mood has a profound influence on cognitive processes. Compared to those in a less positive mood, people in a positive mood tend to have wider visual attention^2^, to activate broader associative memory^3^, and to rely more on general knowledge structures^4^ and stereotypes^5^. Mood also influences the way in which people process language. For instance, people in a good mood (relative to a bad or neutral mood) are more likely to make causality inferences from verbs (termed ‘implicit causality biases’)^6^, sentence context^7^,^8^, and semantic associations in memory^9^,^10^. The syntactic analysis^11^ and the abstract level of autobiographical narratives^12^ are also modulated by mood state, whose effect might further depend on the affective nature of the stimuli^13^,^14^.

The mood effect on language comprehension has been accounted for by two different theories. The first theory, termed the affect-as-information hypothesis^15^, considers mood as a type of information that signals the value of the current processing strategy, which in most cases is engaging in heuristic processing that relates incoming information to what is already known. Positive mood enhances whereas negative mood inhibits this type of information processing, resulting in top-down and bottom-up processing modes, respectively. The other theory takes the perspective that mood directly signals the amount of resources available for exploratory behaviors^16^. A positive mood allows people to invest more energy on external information irrespective of its relevance to the task, whereas a negative mood signals low energy and directs limited resources to relevant/conservative behaviors. In studies where incoming words violated contextual semantic or syntactic prediction^6^,^7^,^8^,^11^, the violation-related ERP effects (e.g. N400 and P600 effects) were smaller in a negative mood than in a positive mood. The reduced ERP effects in a negative mood reflected attenuated expectation on the basis of prior sentential context, which could be due to either less relational processing or limited resources. It therefore remains unclear which mechanism mediates the influence of mood on language comprehension.

The above problem arises because of the lack of a manipulation which can disentangle processing strategy from processing resources. In the above-mentioned studies, the selected processing strategy coincides with the allocation of processing resources. For instance, the positive mood induced associative processing strategy and meanwhile captured sufficient amount of resources, so the semantic violations were easier to detect than in the negative mood^6^,^7^,^8^,^11^. A way to circumvent this is to manipulate the task relevance of stimuli which can be constructed by the linguistic context of sentences. The heuristic processing extracts such contextual associations and makes the best use of them, whereas the analytic processing ignores the context and operates on each component of stimuli. In this sense, the use of heuristic processing strategy would enhance the allocation of attentional resources towards task-relevant stimuli. Based on the “processing strategy” model, task relevant and irrelevant stimuli will be processed differently in a positive mood due to the use of heuristic processing strategy whereas they will be processed similarly in a negative mood due to the use of analytic processing strategy. On the contrary, the “processing resources” model predicts similar processes between task relevant and irrelevant stimuli in a positive mood due to sufficient amount of processing resources but different processes in a negative mood due to limited amount of processing resources. The task relevance of stimuli can be manipulated by a linguistic marker, i.e., information structure (IS). IS refers to the way of packing information^17^,^18^ into focus and background. For instance, “eggplant” in the sentence “*Mum bought **eggplant** for dinner*” is the focused information in the question context “*What vegetables did mum buy for dinner*?”, and is the non-focused information in the context “*Who bought vegetables for dinner*?” Focus can also be used to introduce a set of alternatives (for an overview see ref. 19). In this sense, focus is the most salient information and thus heuristic processing would trigger more detailed processing of the focused information. Studies on the cognitive function of information structure have shown that compared to non-focused, focused words attract more attention and lead to a smaller N400 when they are both congruent in the sentence (for a review see ref. 20). The N400 amplitude has been taken as a reflection of the ease of semantic integration or access^21^. Moreover, semantically incongruent words (e.g. “beef” instead of “eggplant” in the above example) elicit a larger N400 than congruent words, and the effect (i.e., the difference between the two conditions) is larger when target words are focused rather than non-focused^22^. Therefore, IS promotes an attentional strategy to devote more resources to the focused than non-focused information. This enables us to assess the aforementioned mechanisms (“processing strategy” vs. “processing resources”) by examining the respective predictions regarding the processing of focused and non-focused words (Table 1). Based on previous studies^22^, we will focus on the N400 effects in response to semantic incongruence. Specifically, according to the “processing strategy” theory, people in a positive mood allocate their attention primarily to focused words because of their relevance to the current mental setup, thus leading to larger N400 effects; however, people in a negative mood adopt a bottom-up processing strategy and treat the focused and non-focused words equally, leading to comparable N400 effects. On the contrary, the “processing resources” theory predicts that people elaborately process both focused and non-focused words when in a positive mood due to sufficient resources, and allocate limited resources to focused rather than non-focused words in a negative mood. Accordingly, the N400 effects will be comparable in the focus and non-focus conditions with positive mood and be larger in the focus than non-focus conditions with negative mood.

An important concern in studies of mood effects is the mood induction procedure. Previous studies have involved explicit evaluation of films, pictures, music, autobiographic recall and writing tasks (for a review see ref. 23). These methods employed stimuli that were rich in semantic meaning and/or required effortful processing. As a result, cognitive functions other than induced mood might interact with subsequent language tasks. In the current study, we instead used olfactory stimuli to induce mood. Odors are processed by neural structures including the amygdala and orbitofrontal cortices^24^, which are also parts of the limbic system involved in the processing of emotional stimuli^25^ and the regulation of mood state^26^. Consistent with this close anatomical relation, olfactory impairment is regularly found in individuals with affective disorders^27^. Behaviorally, body odors^28^ and ambient environmental fragrances^29^ can alter the mood state of observers, even sometimes without being consciously perceived. Accumulated evidence indicates that pleasant scents tend to have positive effects on mood whereas unpleasant smells tend to have negative effects^30^,^31^,^32^,^33^,^34^. This suggests that odors are natural affective carriers which modulate receiver’s internal state. Because of their robustness in mood induction and virtually effortless processing (i.e., it does not require extra cognitive effort to process), odors have been considered as optimal material in the mood induction procedure^35^. The use of olfactory stimuli thus allows us to attain clear insights into the mechanism of mood effect on language comprehension without obvious confounding influences from other mental processes.

## Results

### Smell and mood ratings

**Figure 1:**
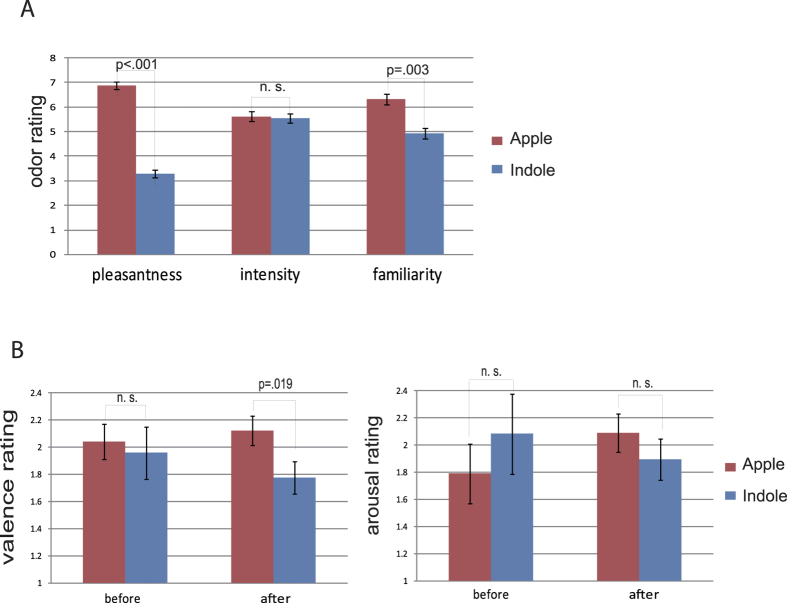
Odor and mood ratings. (**A**) Odor ratings for pleasantness, intensity and familiarity on 9-point scales. The Apple odor was rated to be more pleasant and more familiar than the Indole odor. (**B**) Mood ratings for valence and arousal on −4 to 4 scales. No valence or arousal difference was found before odor application. After odor application, the valence level was higher in the Apple condition than in the Indole condition. Error bars stand for standard error of the mean, adjusted for individual differences.

As shown in Fig. 1A (for the means and standard errors), the apple-like smell (apple flavor) was rated to be more pleasant (*t*_(23)_ = 11.93, *p* < 0.001, 95% CI [−4.20 −2.96]) and familiar (*t*_(23)_ = 3.30, *p* = 0.003, 95% CI [−2.31 −0.53]) but equally intense (*t*_(23)_ = 0.22, *p* = 0.83, 95% CI [−0.87 0.70]) as compared with the dung-like smell (indole). This distinction in perceived valence had a significant effect on the recipients’ mood states. Across six blocks, participants rated themselves, on average, to be happier after exposure to the pleasant odor of apple relative to the unpleasant odor of indole (*t*_(23)_ = 2.53, *p* = 0.019, 95% CI [0.06 0.63]), although their arousal level was similar in these two olfactory conditions (*t*_(23)_ = 1.09, *p* = 0.29, 95% CI [−0.18 0.59]). This mood change was a result of odor exposure, because no significant differences were observed in the baseline state anxiety (Apple session: Mean ± SD = 34.21 ± 8.12; Indole session: Mean ± SD = 32.63 ± 5.90; *t*_(23)_ = 1.11, *p* = 0.278, 95% CI [−1.36 4.53]), trait anxiety (Apple session: Mean ± SD = 40.92 ± 7.31; Indole session: Mean ± SD = 39.75 ± 6.51; *t*_(23)_ = 1.29, *p* = 0.209, 95% CI [−0.70 3.03]), and baseline mood ratings of valence and arousal (valence: *t*_(23)_ = 0.31, *p* = 0.76, 95% CI [−0.47 0.63]; arousal: *t*_(23)_ = −0.62, *p* = 0.54, 95% CI [−1.26 0.68]; also see Fig. 1B) before the odor application.

After the experiment, no participant guessed that the purpose of the experiment was to examine the influence of mood on language processing, although 14 out of 24 participants reported that the smells had affected their mood. It appears that the odor might subtly induce a mood change, and some participants were not consciously aware of such a change. This tendency was confirmed by the small difference between the mood ratings (Apple session vs. Indole session: 2.12 vs. 1.78 on a −4 to + 4 scale). Nevertheless, such faintly induced mood rendered significant consequences on both behavioral and neural responses, as shown below.

### Behavioral results

As shown in Table 2, participants made faster responses to statements following question-answer pairs in the Congruent condition than in the Incongruent condition when they were exposed to the Apple odor (*p*^*2*^ = 0.904, *F*_(1,23)_ = 11.62, *p* = 0.002, 95% CI [−102.38 −25.05]) but not to the Indole odor (*p*^*2*^ = 0.22, *F*_(1,23)_ = 1.51, *p* = 0.232, 95% CI [−117.11 −29.88]). However, participants made more accurate responses in the Congruent than Incongruent conditions when they were exposed to the Indole odor (*p*^*2*^ = 0.75, *F*_(1,23)_ = 7.54, *p* = 0.011, 95% CI [0.014 0.099]) but not to the Apple odor (*p*^*2*^ = 0.33, *F*_(1,23)_ = 2.53, *p* = 0.125, 95% CI [−0.006 0.048]). The different response patterns suggest a speed-accuracy trade-off which is contingent on the smell sampled during the task, with a speed-bias in the Apple condition and an accuracy-bias in the Indole condition.

### ERP results

**Figure 2:**
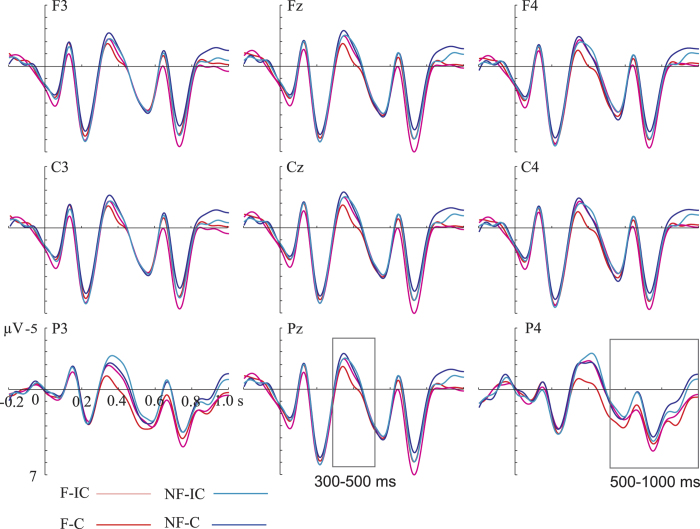
ERPs elicited in the Apple condition. Grand averaged waveforms evoked by the critical words as a function of Context and Congruence at nine representative electrodes. Waveforms are time-locked to the onset of the critical words. Negative is plotted upward. The waveforms were 10 Hz low-band pass filtered for illustrative purposes only. F: focus; NF: non-focus; C: congruent; IC: incongruent.

**Figure 3:**
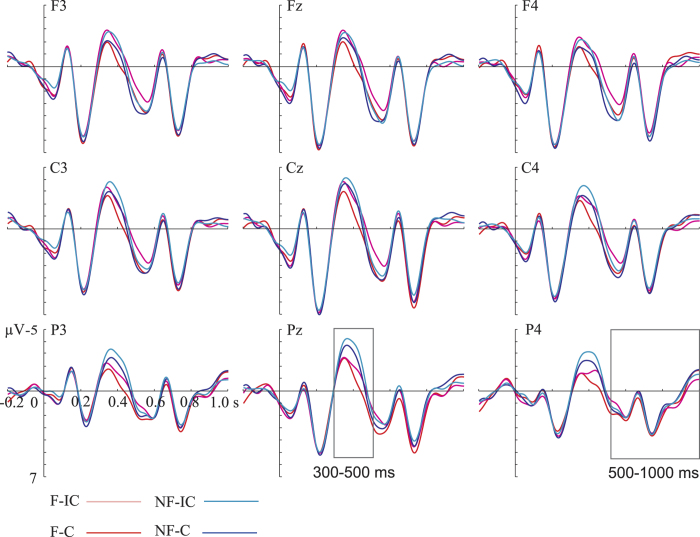
ERPs elicited in the Indole condition. Grand averaged waveforms evoked by the critical words as a function of Context and Congruence at nine representative electrodes. Waveforms are time-locked to the onset of the critical words. Negative is plotted upward. The waveforms were 10 Hz low-band pass filtered for illustrative purposes only. F: focus; NF: non-focus; C: congruent; IC: incongruent.

Figures 2 and 3 present the grand average ERP waveforms evoked by the CWs in the Apple and Indole conditions respectively.

**Figure 4:**
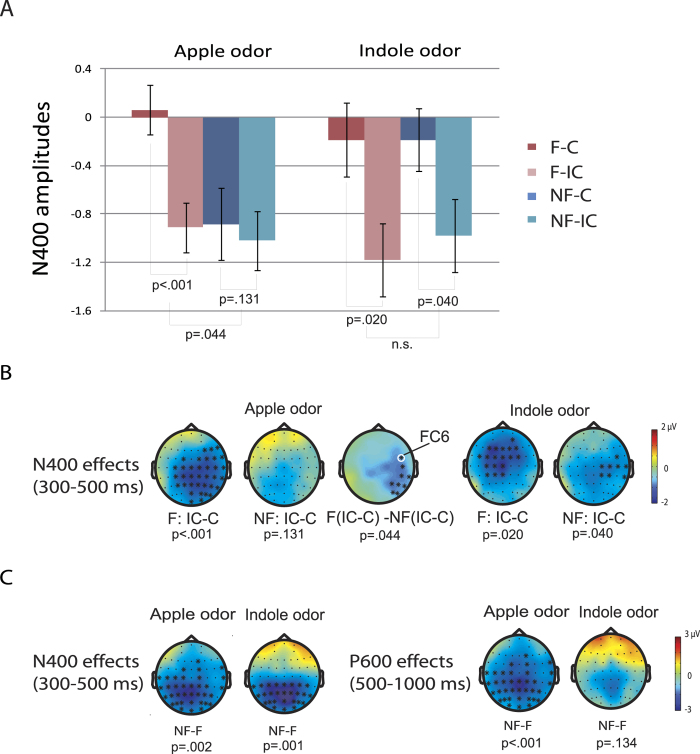
Averaged N400 amplitudes (300–500 ms) in different conditions. Error bars stand for standard error of the mean, adjusted for individual differences. (**B**) Topographic distributions of the Congruence effect in different conditions. The topographies were plotted in the time interval of 300–500 ms. (**C**) Topographic distributions of the Context effect in different conditions. The topographies were plotted in the time intervals of 300–500 ms and 500–1000 ms. The electrodes that showed significant effects are marked as *. F: focus; NF: non-focus; C: congruent; IC: incongruent.

In the Apple condition, there were significant main effects of Congruence (Incongruent >Congruent; *p* = 0.006) and Context (Non-focus >Focus; *p* = 0.002) in the N400 time window. Importantly, the interaction between Congruence and Context in this time window also reached significance (*p* = 0.044) over right frontocentral and posterior region (see Fig. 4B). Simple effects tests showed that the Incongruent words elicited a larger N400 than the Congruent words in the Focus condition (*p* < 0.001) but not in the Non-focus condition (*p* = 0.131). In addition, a marginally significant main effect of Congruence (Incongruent >Congruent; *p* = 0.065) and a significant main effect of Context (Focus >Non-focus; *p* < 0.001) were found in the P600 time window. No significant interaction was found in the P600 time window.

In the Indole condition, there were significant main effects of Congruence (Incongruent >Congruent; *p* = 0.001) and Context (Non-focus >Focus; *p* = 0.021) for the N400, but no interaction between the two factors. For the P600, no significant cluster was found for either the Incongruent vs. Congruent contrast (*p* = 0.166) or the Non-focus vs. Focus contrast (*p* = 0.134). Nor was the interaction between Context and Congruence significant.

Figure 4A summarizes the Congruence effects of N400 amplitudes at electrode FC6 (where the different patterns are representative) under the two olfactory conditions. It is obvious that the effect patterns to Focused and Non-focused CWs are different between the two conditions. The clusters corresponding to the observed Congruence and Context effects are shown in Fig. 4B,C respectively. The observed N400 effects were found over central to posterior regions, which was consistent with the classical N400 effect^21^. The topographic maps of P600 effects showed a posterior distribution, in line with that of previously reported semantic P600 effect^36^.

## Discussion

This study investigated the mechanism through which mood mediates language comprehension. Different moods were successfully induced by odors that differed in pleasantness without obvious changes in arousal level. In the pleasant smell condition, the semantic incongruence evoked a significant N400 effect only in the focus but not non-focus condition. In contrast, the unpleasant smell resulted in significant N400 effects in both the focus and non-focus conditions. These results suggest that the mood state modulates the neural responses involved in the language comprehension and that there exists dissociation between the processing strategies triggered by different mood states. In a positive mood, it seems that a word’s congruency with the previous context is not fully considered when it is not the critical word targeted by the question sentence, whereas in a less positive mood, semantic relations of all words, regardless whether they are critical to the current sentential context or not, are processed up to a certain level which gives rise to the N400 effect. In a more general sense, the current observation implicates that the positive mood encourages an individual to concentrate on the most relevant information in the current context while the less positive mood reminds an individual that details are important so that information is processed analytically and non-selectively. Our findings are thus consistent with the model that regards the mood state as a mechanism to value or devalue the current processing strategy which in most cases is contextual oriented^37^.

We found that participants’ mood was modulated by the exposure of pleasant and unpleasant odors with a higher valence rating in the pleasant than in the unpleasant smell condition (Fig. 1B). It seemed that the mood induction here was conjointly caused by the pleasantness and the familiarity of odors, as demonstrated by a positive correlation between the pleasantness rating and the familiarity rating (r = 0.321, p = 0.026). The respective contributions of pleasantness and familiarity to the mood induction, however, could not be determined in the current setup. It would be interesting to address this concern in future studies by systematically manipulating these two dimensions of odor property. The overall above-zero ratings in both baseline and experimental sessions might be explained by a positive bias when participants rated their valence^38^. We did not include a neutral mood condition, because it is difficult to create (as discussed in ref. 39) and we were interested in the relative rather than absolute mood effects. This procedure, however, limited the generalization of the finding. It is possible that the effect of mood on language comprehension is nonlinear, and a different scenario would occur if a totally negative mood is elicited. Future research is required to address this concern.

Compared with other mood induction methods, the use of smell provides a more implicit way to induce mood with minimal cognitive involvement^35^. In most previous studies, affective films or pictures were evaluated and mood was rated before language tasks^6^,^7^,^8^,^9^,^10^,^11^,^14^, but see ref. 13. Such explicit evaluation might unintentionally invite participants to guess the experimental purpose, leading to the problem of demand characteristics^40^. The current study, on the contrary, showed lack of knowledge in participants of the link between smell and mood or smell and language task, thus largely avoiding such procedural artifacts. Furthermore, evaluating emotional stimuli might also change motivational or alerting levels^6^,^41^. In the current study, odors were task irrelevant thus participants could process the odors passively and presumably effortlessly. Their arousal ratings were also comparable between sessions. We could thus assume with confidence that the motivation and alertness levels were controlled in the current study. Finally, some sensory and perceptual artifacts may also exist in previous studies. For example, visual stimuli with affective tones might differentially activate various visual channels and functions^42^, which potentially influence the reading comprehension^43^. The olfactory system, however, has less direct interaction with low-level processing of written words. Although the exact duration of altered mood induced by odors was unknown, the participants’ mood ratings before and after each block suggest that the pleasant and unpleasant odors induced sustained mood difference between the two conditions. Taken together, the current study adds new data to the existing evidence that olfactory stimuli can be used as optimal material to reliably induce mood in humans^28^,^29^,^30^,^31^,^33^,^34^,^44^,^45^.

We described mood in the current study along the positive-negative axis. As Damasio *et al*.^46^ pointed out, “emotion preceded feeling”, and the general experiences of feeling (perhaps also mood) were pleasure and distress. Nevertheless, moods induced by stimuli in different emotional categories would have discrepant effects on cognitive processes; e.g., a dung-like smell could evoke disgusting emotional responses and induce a negative mood that was different from the negative mood induced by a fearful picture. However, the current study was not designed to differentiate the fine structure of mood and future research is required to address this issue. Considering that mood might be less specific than feeling, we believed that a description along the positive and negative continum could catch the gist of induced moods and thus was appropriate for the current purpose.

Language users selectively process available information during language comprehension^47^ at the expense of failing to notice semantic anomalies in some cases^48^. In the present study, we used IS to guide participants’ selective behavior and used the N400 effect to index semantic integration load due to the mismatch between the incoming and predicted words^21^.

When the participants were in a positive mood state, the semantic incongruence elicited an N400 effect only when the target word was focused, reflecting facilitated semantic integration or access for focused words but limited attentional resources and weakened semantic integration for non-focused words^21^. The dissociation of the N400 effects between focused and non-focused information demonstrates the role of IS on directing resources for semantic processing (for a recent review see ref. 20) and suggests that people actively use IS during language processing when they are in a positive mood. In addition to the N400 effect, the semantic incongruence evoked (marginally significant) P600 effects for both the focused and non-focused words. This is not necessarily inconsistent with the preceding N400 pattern, as the P600 effect has been associated to word re-analysis, especially when people temporarily fail to detect a violation^49^,^50^. Therefore, the P600 effects for both focused and non-focused words suggest that people might eventually detect the semantic incongruence even after an initial failure–indicated by a lack of N400 effect–for the non-focused words due to the use of heuristic processing strategy.

When the participants were in a less positive mood, the semantic incongruence elicited comparable N400 effects in both the focus and non-focus conditions. The reduced N400 amplitude for the C words compared to the IC words (i.e., the N400 effects in response to the semantic incongruence) suggests that words, regardless of IS, in the indole condition received sufficient attentional resources so that their semantic relationships were processed to a certain level leading to N400. Therefore, the effectiveness of IS on language processes was diminished in a relatively less positive mood.

Moreover, compared with non-focused words, focused words elicited a larger P600 in a positive mood but a comparable P600 in a less positive mood. Although the functional interpretation of the P600 effect remains controversial (for a review see ref. 20), the larger P600 amplitude has been related to extra allocation of attentional resources^51^, or prolonged analysis of linguistic inputs^52^, potentially supporting different processing strategies observed in N400 effects between positive and less positive conditions. In a relevant study^53^, attention was directed to syntactic or physical features of sentences. The results showed that syntactic violations elicited reduced P600 effects regardless of attention in a negative mood, but a larger P600 effect when attention was directed to (vs. away from) the syntactic features in a positive mood. Although the syntactic P600 in this study might be different from the semantic P600 in the current study^36^, the observation of Verhees *et al*.^53^ suggests that different reliance on heuristic processing might lead to mood-related differences in language processes.

What is the underlying mechanism by which mood modulates language comprehension? Based on the predictions listed in the *Introduction*, current results support the “processing strategy” hypothesis in line with the “affective-as-information” theory^54^. When participants were in a positive mood, they implemented a heuristic strategy that relied on IS to effectively allocate their processing resources to the most important (focused) information, with limited resources allocated to the irrelevant (non-focused) information. When they were in a less positive mood, their processing was less guided by IS, resulting in non-selective semantic analysis of both focused and non-focused information. In support of this hypothesis, a speed-accuracy trade-off was found in the responses to the statements as a function of mood (Table 2). When in a positive mood, participants made responses based on the general information obtained from preceding question-answer pairs, leading to faster responses for the statements matching the question-answer pairs. However, when participants were in a less positive mood, they were guided by a more analytic processing mode, and the difference between congruent and incongruent pairs was expressed more in the response accuracies. Besides, the overall response pattern also exhibited different biases to the speed and accuracy, with the apple condition showing faster responses and the indole condition showing higher accuracy. This result also renders the possibility of impaired processing of IS in the indole condition unlikely. As introduced in the beginning, IS in the current study refers to the packaging of focused and non-focused information in the answer sentence under different question contexts. Thus the reduced efficiency of IS processing would most likely result in impaired semantic processing of the question-answer pairs. However, the behavioral performance did not show a significant semantic processing impairment that could reveal substantial reduction of IS processing in the indole condition. For example, the accuracy in the indole condition was higher than that in the apple condition; the RT difference was only on the order of dozens of milliseconds delay, which could be considered negligible in the context of much longer presentation of question sentence (3000 ms) and answer words (300 ms each). Of course, the current study could not exclude the above possibility completely, and more direct evidence is definitely required to either reject or accept it. The modulation of processing strategy by mood has received support from other studies. It has been shown that people are more likely to make predictions based on previous context in a positive mood condition, eliciting larger ERP effects when they read unexpected words when in a positive rather than negative mood^6^,^7^,^8^. People have also had broader semantic associations when in a positive rather than negative mood, as indicated by the different processing of semantically unexpected words that are nevertheless semantically related to the highly expected words in different mood conditions^9^,^10^. Moreover, in a description of social events, positive mood gave rise to the use of more abstract linguistic expressions because of the global processing style, whereas negative mood resulted in more concrete descriptions due to the detail-oriented analytic processing style^12^. Furthermore, outside the language domain, relative to negative mood, positive mood tends to widen the scope of visual attention^2^, to increase the scope and impact of routine memory-based thinking^3^, and to make people more susceptible to misinformation^55^.

Although the previous studies could also be accounted for by the “processing resources” hypothesis, the present data clearly argue against this hypothesis. The “processing resources” hypothesis proposes that mood modulates the effort invested in cognitive processing, with higher energy and more processing resources available in a positive than negative mood. This hypothesis was derived based on a bio-energetic principle^16^. It has been shown that people in a negative mood overestimate the steepness of a hill and the distance from a balcony to the ground^16^. In this vein, a positive mood endows the neural system to distribute resources unselectively to multiple stimuli, whereas a negative mood constrains the system to focus only on the most relevant items. Results of the present study show, however, the opposite pattern. That is, participants selectively allocate their attention to task-relevant stimuli (i.e., the focused words) when in a positive mood, whereas they distributed their attentional resources to both relevant and irrelevant stimuli in a less positive mood. One might also argue that participants in the indole condition used more, rather than less, resources so that both the focused and non-focused words were sufficiently processed. This possibility is inconsistent with both hypotheses described above. If more resources were indeed available in the indole condition, one would also expect that the responses to statements in the indole condition compared with the apple condition were more accurate and faster. However, the behavioral results (Table 2) showed a trade-off between the accuracy and speed, not fully supporting this hypothesis. Nevertheless, without direct measurement of overall resources the current study could not completely exclude this possibility, and further research is needed to systematically assess its plausibility.

In addition to the difference of induced mood, the odors also differed in pleasantness and familiarity. To check whether the mood instead of the odor properties accounts for the N400 effect, we performed partial correlation analyses between the differences (Apple–Indole) in the mood ratings (including valence and arousal) and the difference (Apple–Indole) of the observed N400 effects (Focus (IC-C)–Non-focus (IC-C)), with the odor ratings (including pleasantness and familiarity) serving as control factors. The analyses were performed at electrodes that showed significant interaction effects (Context × Congruence) in the Apple condition (i.e., electrodes FC6, C6, CP6, TP8, P4, P6, P8, PO6, PO8). We found significant correlations between the N400 effect and the mood ratings at both FC6 (r = −0.546, p = 0.009; r = −0.545, p = 0.009 respectively for the valence and arousal ratings) and C6 (r = −0.438, p = 0.041; r = −0.532, p = 0.011 respectively for the valence and arousal ratings). Although after the Bonferroni correction the significance level of the above analysis did not reach the criterion of 0.05 and some caution has to be taken for the interpretation of the result, the partial correlation analysis provides additional evidence on the mood’s influence on the N400 effect. This also echoes to the situation that participants in our study concentrated on the language task and the irrelevant odors did not have any direct semantic or object-related connection with the language material. It is thus unlikely that the odor properties of pleasantness and familiarity had ever been transferred to the language stimuli via their implicit meaning associations. One could further argue that participants might have attempted to label the odors during the experiment. Compared with the pleasant odor, the unpleasant odor was unfamiliar to participants, thus the labeling process was more difficult. This might create an ambiguity in the categorization system which would, perhaps partially, reduce the usage of the heuristic strategy, consistent to the “processing strategy” hypothesis. The labeling difficulty also might command more attentional resources to label the unpleasant odor while leaving fewer resources available for the language task. If this was the case, we would have observed smaller N400 effects in the unpleasant odor condition, which was contrary to the results. Moreover, it has been shown that mixture of monomolecular odors (e.g., the apple odor) tends to disrupt linguistic processing compared to monomolecular odors (e.g., the indole)^56^. If this was the case, the N400 effects for the focused words in the pleasant and unpleasant odor conditions would be significantly different. However, our results showed comparable N400 effects between the two odor conditions for the focused words. Therefore, the observed effects in the current study are most likely explained by the induced moods following pleasant and unpleasant odor exposure.

## Conclusions

This study examined the mechanism through which odor-induced mood modulates language comprehension. In a positive mood, semantically incongruent relative to congruent words evoked larger N400s when they were focused but comparable N400s when they were non-focused. In contrast, in a less positive mood, congruence-related N400 effects were similar between focused and non-focused conditions. These results indicate that different processing strategies are employed in different mood states, with heuristic control of sentential context to direct resources to relevant signals in a positive mood and parallel bottom-up processing of multiple inputs in a less positive mood. Our findings have ecological implications for language comprehension. Mood might signal the environmental situations in a way that positive and negative moods indicate safe and dangerous environments, respectively. Therefore, only the most important information is considered to be worthy of detailed processing when people are in positive mood whereas language comprehenders will take all information as being important when they are in a less positive mood.

## Materials and Methods

### Participants

Twenty four female university students (mean age 22 years, 19–26 years old) served as paid volunteers. Only females were recruited because they have an overall superior sense of smell^57^ and are more sensitive to mood induction^9^ than males. All were right-handed native speakers of Chinese with self-reported normal or corrected to normal vision and normal sense of smell. None of them was smoker and alcoholic. None had dyslexia or any neurological impairment, or reported respiratory allergy or upper respiratory infection at the time of testing. They were not menstruating at the time of participation. All experimental protocols were approved by the Institutional Review Board of the Institute of Psychology, Chinese Academy of Sciences and all participants were treated in accordance with the Declaration of Helsinki. Written informed consent was obtained from all participants. Methods were carried out in accordance with the approved guidelines.

### Stimuli

Experimental materials included visual stimuli presented in the form of question-answer pairs and olfactory stimuli with different valences. In each visually presented question-answer pair, the question established a context that projected a focus position in the answer sentence. In the *what-kind-of*- question context the critical word (CW) was a focused word, whereas in the *who*- question context the CW was a non-focused word (see examples in Table 3). In addition, the CW in the answer sentence was either congruent or incongruent with the question context (Table 3). The CWs were never in the sentence-initial or sentence-final position. Using a full factorial design for the CWs with factors of Context (Focus, Non-focus) and Congruence (Congruent, Incongruent), four conditions were formed for each item set. The congruent and incongruent words were matched on log-frequency (mean ± SD = 1.80 ± 1.00; 1.92 ± 1.00, respectively) based on a Chinese corpus developed by^58^: *t*_(319)_ = −1.73, *p* = 0.085. It should be noted that the congruence effects (i.e., the contrast between the Congruent and Incongruent words) were identical between the Focus and Non-focus conditions, so the word properties of the Congruent and Incongruent words had no effect on the comparison of congruence effects between Focus and Non-focus.

The four conditions of the 320 item sets were distributed across four experimental lists according to a Latin square procedure, with each list containing equal number of items (80 items) per condition. In order to make the experimental materials non-transparent to the participants, we constructed 160 fillers, with *what*- or *who*- question context in each half of the fillers (Table 3). No sub-categorical information was required in the fillers with *what*- question context while no extra information was present in the fillers with *who*- question context. All the fillers were semantically congruent and were added to the four lists. Consequently, there were 480 items in each experimental list (320 experimental items and 160 filler items). Each list was further divided into two sessions, with each session containing the same number of items per condition. Therefore, each session contained 240 items (160 experimental items and 80 filler items), with the same number of items (40 items) per condition (four conditions in total) for the experimental items. The sets of stimuli were balanced but not identical across odor-sessions. The four lists were equally distributed across the 24 participants.

Olfactory stimuli were apple flavor (Givaudan) and indole diluted by propylene glycol with concentration of 0.02% v/v and 0.025 g/ml, respectively. The former smells like apple juice (a pleasant smell) whereas the latter smells similar to animal dung (an unpleasant smell), thus conveying positive and negative valences, respectively. There was no semantic association between the two odors and any specific language stimulus. Odorants were sampled by participants via two Teflon nosepieces attached to a Y structure which in turn was connected to a 280 ml bottle. Bottles containing olfactory stimuli were identical and both odor solutions were 10 ml clear liquids. Thus no visual signals communicated valence information to the participants.

### Procedure

Participants were seated in a comfortable chair in front of a CRT monitor (DELL Trinitron P1130) which has a screen size of ~38.5 × 29 cm and a resolution of 1024 × 768 pixels. Stimulus presentation and data collection was controlled by a PC running Windows system and E-Prime software (Psychology Software Tools Inc., Pittsburgh, PA). On arrival, participants first signed a written consent form before filling in the Spielberger State and Trait Anxiety Assessment Inventory (STAI)^59^ which measures state anxiety and trait anxiety with 20 questions, each based on a 4-point Likert scale. After a short practice session consisting of eight question-answer pairs, participants were asked to rate their current mood in terms of valence and arousal by pressing labeled numbers on a keyboard. The valence scale ranged from “extremely sad” (−4) to “extremely happy” (+4) (with “neutral” at 0), and the arousal scale ranged from “extremely calm” (−4) to “extremely exited” (+4) (with “neutral” at 0). Both the STAI and the mood ratings were taken as a baseline measure of their mood before olfactory exposure. After this, the participants were asked to familiarize themselves with the olfactory stimulus during electrode placement and skin preparation. The familiarization time was comparable across participants and conditions (around 30 minutes).

The language stimuli were presented in font size 18 as white characters on a black background in the center of the screen, positioned approximately 80 cm away from the participants. A trial started with a fixation cross (duration 2000 ms) in the center of the screen, followed by a question that was presented as a whole sentence for 3000 ms. After a 200 ms black screen, the answer was presented word by word, with each word appearing for 300 ms, interleaved by an interstimulus interval (ISI) of 200 ms. Participants were told not to move their eyes or blink when individual words appeared. To ensure that participants read for comprehension, they were required to judge the correctness of a statement following the answer sentence by pressing one of two buttons in one third of trials. The statement referred to the semantic content of the answer but not the semantic relationship between the question and the answer (e.g., the statement following the example experimental item in Table 3 was “*Xiaoli is good at sports*.” and was correct). In the remaining trials, there were no statements and participants were instructed to press a third button. All responses were demanded within a window of 4000 ms after the offset of the answer sentence. During the presentation of the question-answer pairs, participants were continuously exposed to one of the olfactory stimuli. They were instructed to inhale through the nose and exhale through the mouth throughout each block, which lasted approximately six minutes. Between blocks, there was a 2–5 minute rest period during which no odor was presented. Participants were asked to rate their mood in terms of valence and arousal before and after each experimental block.

The 480 items in each list were divided into 12 blocks (40 trials per block), which were further separated into two sessions (6 blocks per session). Participants were exposed to the pleasant smell of apple in one session and the unpleasant smell of indole in the other. The two sessions, whose order was counterbalanced across participants, were performed 3–7 days apart. Each session took approximately two hours, including preparation, the language task, and an unrelated spatial attention task which lasted approximately 15 minutes. After each session, the participants rated the smell in terms of pleasantness, intensity and familiarity on 9-point scales, with 9 indicating the most pleasant, intense or familiar. Participants were also asked to guess the purpose of the experiment and to reflect on whether the odor had affected their mood after they had finished the whole experiment. Information from this post inquiry can provide some clues of the demand characteristics in the experiment.

### Electroencephalogram (EEG) recording and analysis

The EEG data were recorded by a 64-channel NeuroScan system with electrodes positioned according to the extended 10–20 system. The left mastoid electrode served as the reference, and an electrode placed between the Fz and Fpz electrodes served as the ground. Vertical (VEOG) and horizontal (HEOG) eye movements were monitored by four electrodes around the orbital region (bipolar montage). Electrode impedances were kept below 5 KΩ and online data were band-pass filtered (0.05–100 Hz) and sampled at 500 Hz.

The data were re-referenced off-line to the average of both mastoids and filtered with a 0.1–30 Hz (24 dB/oct slope, half-power cutoff) band-pass filter. Ocular artifacts were automatically corrected by NeuroScan software^60^. Data epochs were extracted from −0.2 s to 1.0 s relative to the onset of the CWs. Trials with EEG amplitudes exceeding ±80 μV or with incorrect responses were discarded before averaging for ERPs. On average, there were 37 trials (36–37 trials) in each condition, with similar numbers across conditions.

### Statistical analysis

ERP differences between conditions were statistically evaluated in Fieldtrip^61^ by cluster-based random permutation tests each with 1000 times of random permutation^62^. This approach controls Type-I error rate, which is inflated by traditional multiple comparisons across electrodes. On the basis of earlier studies^22^ and visual inspection (see Figs 2 and 3), N400 and P600 were defined in time windows of 300–500 ms and 500–1000 ms, respectively. ERP signals were averaged across each time window before entering statistical tests to increase the signal-to-noise ratio. Though framed for pair-wise comparisons, the permutation test can accommodate a 2 × 2 experimental design. The main effect of Congruence was tested by comparing the amplitude of the Incongruent condition (averaging across Focus-Incongruent and Non-focus-Incongruent conditions) with that of the Congruent condition (averaging across Focus-Congruent and Non-focus-Congruent conditions). Similarly, the main effect of Context was obtained by comparing the amplitude of the Non-focus condition (averaging across Non-focus-Congruent and Non-focus-Incongruent conditions) with that of the Focus condition (averaging across Focus-Congruent and Focus-Incongruent conditions). Subsequently, the interaction between Context and Congruence was assessed by comparing two subtractions: (Focus-Incongruent minus Focus-Congruent) vs. (Non-focus-Incongruent minus Non-focus-Congruent). If the interaction was significant, further simple effects analyses were conducted. Since we have an a priori hypothesis that language processes would differ under different odor-induced mood states, we separately tested the aforementioned effects for the Apple and Indole conditions.

## Additional Information

**How to cite this article**: Wang, L. *et al*. Odor-induced mood state modulates language comprehension by affecting processing strategies. *Sci. Rep*. **6**, 36229; doi: 10.1038/srep36229 (2016).

**Publisher’s note:** Springer Nature remains neutral with regard to jurisdictional claims in published maps and institutional affiliations.

## Figures and Tables

**Table 1:** Predictions of the N400 effect in response to semantic incongruence based on the two mechanisms triggered by mood.

Possible mechanisms	Positive mood	Negative mood
Processing strategy	Rely on information structure: F > NF	Rely on bottom-up input: F = NF
Processing recourses	Sufficient resources for both F and NF: F = NF	Insufficient resources for NF: F > NF

Notes: F: focus; NF: non-focus.

**Table 2:** Behavioral results for the statements following question-answer pairs.

Conditions	F-C	F-IC	NF-C	NF-IC
RT: Apple	1424.40 (360.08)	1500.55 (383.74)	1437.55 (360.37)	1488.82 (359.56)
RT: Indole	1474.54 (318.53)	1530.14 (304.74)	1515.96 (347.64)	1547.59 (307.04)
ACC: Apple	89.58 (9.08)	88.33 (9.17)	88.33 (9.63)	85.42 (9.77)
ACC: Indole	92.08 (8.33)	86.25 (10.14)	92.50 (8.97)	87.08 (11.97)

Note: The mean (standard deviation) values are shown in the cells. RT: response time; ACC: Accuracy; F: Focus; NF: Non-focus; C: Congruent; IC: Incongruent. The RT is shown in milliseconds and the accuracy is calculated as a percentage.

**Table 3:**
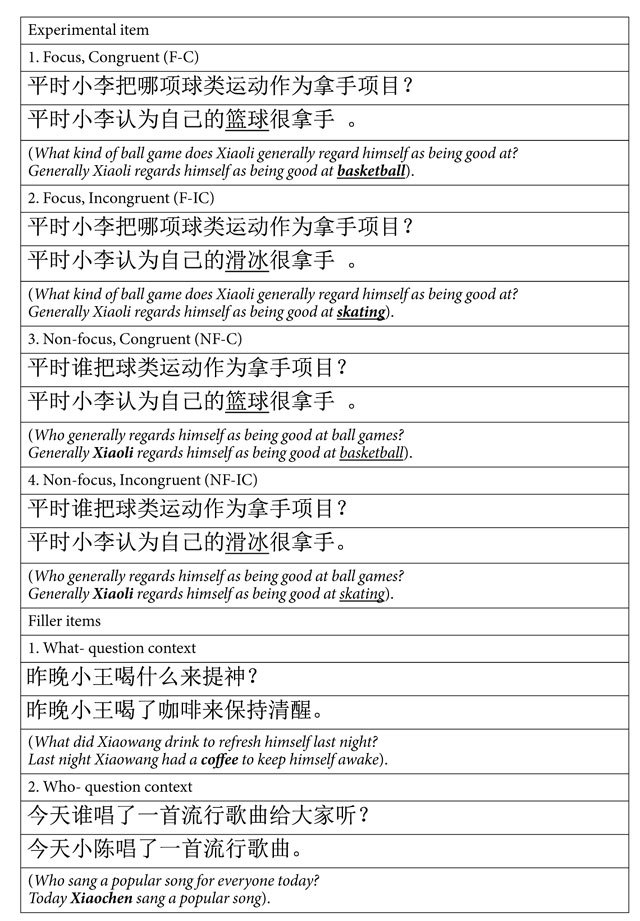
Examples of one experimental item and two fillers.

Note: The stimuli were originally in Chinese, and the English translations are given in the table. The critical words are underlined, and the focus of the sentences was in bold.
